# Determinants of postpartum uterine atony in urban South Ethiopia: a community-based unmatched nested case–control study

**DOI:** 10.1186/s12884-023-05820-1

**Published:** 2023-07-06

**Authors:** Belayneh Hamdela Jena, Gashaw Andargie Biks, Yigzaw Kebede Gete, Kassahun Alemu Gelaye

**Affiliations:** 1 Department of Epidemiology, School of Public Health, College of Medicine and Health Sciences, Wachemo University, Hossana, Ethiopia; 2 Department of Health System and Policy, Institute of Public Health, College of Medicine and Health Sciences, University of Gondar, Gondar, Ethiopia; 3 Department of Epidemiology and Biostatistics, Institute of Public Health, College of Medicine and Health Sciences, University of Gondar, Gondar, Ethiopia

**Keywords:** Uterine atony, Determinants, Nested case–control study

## Abstract

**Background:**

Uterine atony is the most common cause of postpartum hemorrhage, which is the leading preventable cause of maternal morbidity and mortality. Despite several interventions uterine atony-related postpartum hemorrhage remains a global challenge. Identifying risk factors of uterine atony helps to reduce the risk of postpartum hemorrhage and subsequent maternal death. However, evidence about risk factors of uterine atony is limited in the study areas to suggest interventions. This study aimed to assess determinants of postpartum uterine atony in urban South Ethiopia.

**Methods:**

A community-based unmatched nested case–control study was conducted from a cohort of 2548 pregnant women who were followed-up until delivery. All women with postpartum uterine atony (*n* = 93) were taken as cases. Women who were randomly selected from those without postpartum uterine atony (*n* = 372) were taken as controls. Using a case to control ratio of 1:4, the total sample size was 465. An unconditional logistic regression analysis was done using R version 4.2.2 software. In the binary unconditional logistic regression model variables that have shown association at *p* < 0.20 were recruited for multivariable model adjustment. In the multivariable unconditional logistic regression model, statistically significant association was declared using 95% CI and *p* < 0.05. Adjusted odds ratio (AOR) used to measure the strength of association. Attributable fraction (AF) and population attributable fraction (PAF) were used to interpret the public health impacts of the determinants of uterine atony.

**Results:**

In this study, short inter-pregnancy interval < 24 months (AOR = 2.13, 95% CI: 1.26, 3.61), prolonged labor (AOR = 2.35, 95% CI: 1.15, 4.83), and multiple birth (AOR = 3.46, 95% CI: 1.25, 9.56) were determinants of postpartum uterine atony. The findings suggest that 38%, 14%, and 6% of uterine atony in the study population was attributed to short inter-pregnancy interval, prolonged labor, and multiple birth, respectively, which could be prevented if those factors did not exist in the study population.

**Conclusions:**

Postpartum uterine atony was related to mostly modifiable conditions that could be improved by increasing the utilization of maternal health services such as modern contraceptive methods, antenatal care and skilled birth attendance in the community.

**Supplementary Information:**

The online version contains supplementary material available at 10.1186/s12884-023-05820-1.

## Background

Uterine atony is defined as inadequate contraction of the uterus during labor and after delivery of the baby [1]. Normally, uterine muscles (myometrium) contract during labor to deliver the baby, and immediately after delivery to expel the placenta in response to endogenous oxytocin that is released in the course of delivery [2]. Contraction of uterine muscles compresses the blood vessels, which slows blood flow, and promotes coagulation. These processes help prevent postpartum hemorrhage which is a leading cause of maternal morbidity and mortality, globally [3–5].

Uterine atony is one of the most common causes of postpartum hemorrhage, which accounts for up to 80% of cases [6]. Other causes, such as retained placenta, placenta accreta, vaginal tears or lacerations, and uterine rupture, can lead to postpartum hemorrhage [7, 8]. The magnitude of uterine atony and risk factors are poorly understood and vary from setting to setting [9]. In the United States, uterine atony occurs in 1 in every 40 births, or 2.5% [10]. In Madagascar, it occurs in about 0.73% of births [11].

Risk factors of uterine atony include but are not limited to prolonged labor, uterine distension (often due to multifetal gestation, polyhydramnios, fetal macrosomia), chorioamnionitis, indicated magnesium sulphate infusion, prolonged use of oxytocin, fibroid uterus, retained placental products, placental disorders such as placenta previa, adherent placenta, placental abruption, uterine inversion, coagulopathy, body mass index (BMI) above 40 kg/m^2^ and multi-parity [1, 3, 10, 12, 13]. These factors are more clinical and linked to each other. Previous studies reported factors associated with either atonic postpartum hemorrhage [3] or just postpartum hemorrhage [14–16]. Studies that identify distal factors, such as socio-demographic and reproductive characteristics, associated with uterine atony are limited, especially in the study setting.

According to the World Health Organization's (WHO) recommendation, a number of procedures were being used to treat uterine atony-related postpartum hemorrhage, including uterotonics, tranexamic acid, and intravenous fluid with isotonic crystalloids, which are first-line treatments [3, 17]. Some women with those treatments still continue to bleed (refractive postpartum hemorrhage) and need further treatments. Bimanual uterine compression, external aortic compression, non-pneumatic anti-shock clothing, uterine balloon tamponade, and a second dose of tranexamic acid are all suggested by the WHO as nonsurgical interventions to treat refractory postpartum hemorrhage, with a focus on low-resource settings where operating rooms are not always accessible [3, 17, 18]. Despite these interventions and emphasis, uterine atony-related postpartum hemorrhage remains a problem globally, particularly in developing countries [6, 7].

Uterine atony is a difficult condition to predict, and risk factors may vary from setting to setting [3, 19]. The findings from this study can provide additional input about the risk factors of uterine atony for evidence-based decision-making. Therefore, the aim of this study was to assess the determinants of postpartum uterine atony in urban South Ethiopia.

## Methods

### Study design and setting

In this study, a community-based unmatched nested case–control study design was applied. The cases and controls originated from a community-based prospective cohort study that was conducted among pregnant women in five urban settings: namely Hossana, Shone, Gimbichu, Jajura, and Homecho, which are located in Hadiya Zone, South Ethiopia. Hadiya Zone is located 232 km from the capital city, Addis Ababa. In the zone, there are 1 general hospital, 3 primary hospitals, 62 health centers, and 311 health posts that provide health services for the community (Hadiya Zone Health Bureau report, unpublished).

### Participants

Study participants involved in this study were subsets of a community-based prospective cohort study that was conducted to assess the effects of inter-pregnancy intervals on pregnancy outcomes [20]. For the study, a cohort of pregnant women who were at the end of their first trimester of confirmed pregnancy (after 12 weeks of gestation) were enrolled via house-to-house visits, from July 8, 2019, to March 30, 2020. Women who were pregnant at the time of recruitment, who were able to recall the date of last childbirth, who had a live birth during the most recent childbirth, who had no recent stillbirth, and who had no recent abortion were included. In the study, a total of 2578 pregnant women were enrolled and followed until September 30, 2020 [20].

For this particular study, all participants who had experienced uterine atony during a follow-up time (July 08, 2019 to September 30, 2020) were considered as cases and those who had no uterine atony were considered as controls.

### Sample size

**Fig. 1:**
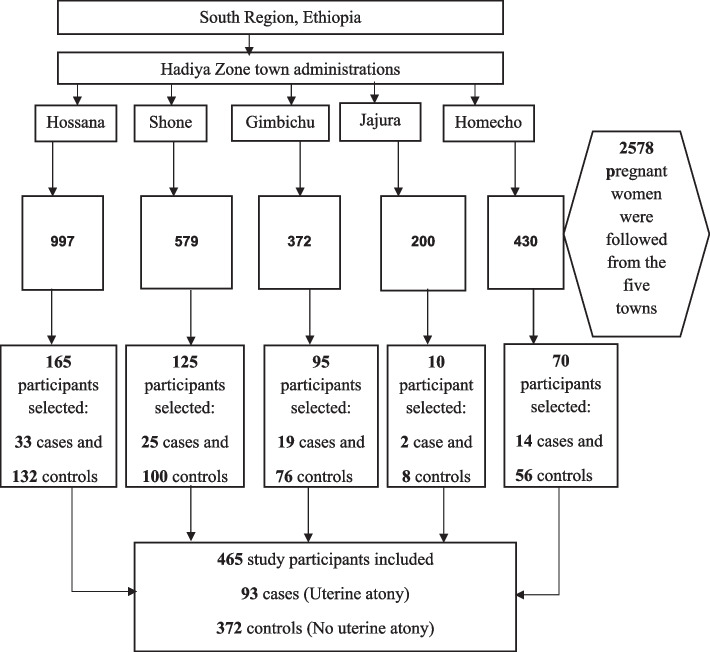
Schematic presentation of sampling in urban South Ethiopia, July 2019-September 2020

In nested case–control studies, the sample size depends on the number of observed cases from a cohort study and their corresponding controls. Accordingly, a total of 93 cases were observed during the follow-up. Uterine atony is a relatively rare condition. To increase statistical power (precision), a 1:4 ratio was considered. Therefore, the sample size becomes 465 (93 cases and 372 controls). For every uterine atony case, four controls were randomly selected from the frame of the cohort (the risk set) by using a random number generator in open-Epi software. The sample frame was prepared for each study site separately so that for a given case, the corresponding four controls were selected from where the case was obtained (Fig. 1).

### Variables and measurements

#### Dependent variable

The dependent/outcome variable was uterine atony. Uterine atony is usually diagnosed during a physical examination immediately after delivery. An examination to check uterine tone after cesarean delivery commonly involves direct palpation of the uterus. After vaginal delivery, an indirect bimanual examination can be used to check uterine tone. The examination reveals a uterus that seems enlarged and soft (boggy) if uterine atony happens. The uterus usually contains a significant amount of blood in its cavity, and bleeding via the vaginal canal is common [10]. For this study, we used clinically diagnosed uterine atony reported in clients’ charts. Then uterine atony was categorized as a binary variable and coded as ‘0’ and ‘1’ (0 = absent (controls) and 1 = present (cases)).

### Independent variables

Independent variables were: Socio-demographic, economic and reproductive characteristics, which are measured as follows: The age of a woman is measured in years by asking how old she is and when she was born. Age at first childbirth measures at what age she has given her first birth, irrespective of the outcomes of the birth, and is reported in completed years. Parity is measured by asking a woman the number of times she gives birth, irrespective of the birth outcomes. The response was reported in numbers, and categorized as prim-para (if she has given birth only once) or multipara (if she has had more than one birth). The inter-pregnancy interval is measured by asking women about the dates of their most recent childbirth and their last menstrual period. Then it was computed by subtracting the date of recent childbirth from the date of the last menstrual period (LMP). For women who had difficulty recalling the date of LMP, ultrasound was used to estimate gestational age at the hospitals. LMP was computed by subtracting the duration of gestation, and then the value of the inter-pregnancy interval was calculated. The inter-pregnancy interval was categorized as < 24 months (short) and 24–60 months (normal), which is based on the World Health Organization recommendation for birth spacing [21]. Pregnancy intention refers to whether the woman had a plan for pregnancy at the time of conception. If a woman had a plan, the response was categorized as ‘intended; if not, it was categorized as 'unintended'. Unintended pregnancy includes both mistimed and totally unwanted pregnancies [22]. Pre-eclampsia was measured as the presence of pregnancy-related high blood pressure (≥ 140/90 mmHg) and protein in the urine, after 20 weeks of gestation [23], and was taken from the clients’ chart. If it occurs, it is reported as "present," otherwise it is reported as "absent." Premature rupture of membranes was measured as a rupture of membranes before the onset of labor and is categorized as ‘present’ or ‘absent [24]. The duration of labor is the time that a woman stays in labor. If the true labor exceeds 12 h, irrespective of the stages, then it is categorized as ‘prolonged labor, otherwise ‘not prolonged' [25]. The progress of labor refers to whether labor progresses in its natural course or is augmented or induced using utero-tonic drugs. If labor was induced or augmented, then it was classified as ‘assisted’, otherwise 'normal'. The mode of delivery is whether a woman gives birth spontaneously vaginally, assisted vaginally with instruments (forceps and vacuum), or via cesarean section. Birth characteristics refer to the number of babies born at the time of delivery (single or multiple). Gestational age refers to the number of weeks elapsed from the date of a woman’s last menstrual period until the date of delivery. If it was < 37 completed weeks, it was categorized as ‘preterm’, if it was from 37–41 weeks, including the 37^th^ week, it was categorized as ‘term’, and if it exceeded 41 weeks, it was categorized as 'post-term' [26]. Birth weight is the weight of the newborn at birth, measured in grams. It was categorized as < 4500gms and ≥ 4500gms (macrosomia) [27].

### Data collection procedures

The questionnaire was prepared in English from existing related literature (published articles and Ethiopia Demographic and Health Surveys) based on the study objectives [28, 29]. The English version was translated to Amharic by two native speakers of the Amharic language (one was public health and the other was English language and literature in their professions). Then back translation to English was done by another two individuals who could speak English (again, one was from public health and the other from English language and literature). The questionnaire was pre-tested on 50 pregnant women in Durame town, where the actual study population is culturally related.

Baseline data about sociodemographic and reproductive variables were collected at the household level during enrolment via face-to-face interviews. Ten trained midwives collected data and five public health professionals made supervisions. The data collectors at each health facility were assigned and the list of participants was given for each of them to collect outcome data. The outcome (uterine atony) and other clinical data were collected from clients’ charts, during the time of delivery before discharge [20].

### Analysis

Data were entered in Epi-data version 3.1 software and exported to R version 4.2.2 software for the analysis. For continuous variables, mean and standard deviation were calculated. For categorical variables, frequencies and percentages using cross-tabulation were calculated. For missing data, a complete case analysis approach was applied. Interactions for possible effect modifications were checked for the predictor variables. To identify determinants of uterine atony, an unconditional binary logistic regression analysis was done. All independent variables that showed a significant association with uterine atony in the binary unconditional logistic regression model at *P* < 0.20 were included in the multivariable unconditional logistic regression model. Then predictor variables with confidence intervals for odds ratios that did not include 1 and *P* < 0.05 were declared statistically significant determinants of uterine atony. To measure the impact of the determinants, attributable fractions (AF) and population attributable fractions (PAF) were estimated using the adjusted odds ratios (AOR) and percent of cases exposed as follows: AF = [(AOR-1)/AOR]*100; PAF = AF*% of cases exposed. Percent of cases exposed = the number of exposed individuals among those who had an outcome divided by the total number of individuals with the outcome multiplied by 100 [30, 31].

### Quality control measures

Two days training was given for data collectors and supervisors on the concept of the questionnaire and how to approach the participants ethically. A pre-test was conducted in Durame town, which has a similar socio-cultural context to the study setting. Supervisors checked the data collection process closely. To minimize selection bias, we used community-based recruitment to include pregnant women during the study period using predefined eligibility criteria. Additionally, ultrasound was used for those women who had difficulty remembering the date of their last menstrual period due to different reasons, such as contraceptive use and breastfeeding. Epi-data was used to control errors during data entry. The data were explored to check for outliers and missing values.

## Results

### Cohort information

A total of 2578 pregnant women from the end of first trimester were followed-up until delivery. Of these, 29 (1%) were lost to follow-up, and their pregnancy outcomes could not be ascertained. The pregnancy outcomes were ascertained for the remaining 2549 study participants. One woman had a spontaneous abortion before 28 weeks of gestation. Hence she was not followed-up any more. The final analysis was done for the remaining 2548 study participants. Of them, 93 developed uterine atony, yielding the incidence of uterine atony 3.7% (Additional Fig. 1).

### Socio-demographic, economic and reproductive information

In this study, data were missing for age (*n* = 3) and age at first childbirth (*n* = 1). Missing data were not related to the outcome (uterine atony) since the missed variables were collected at baseline before the outcome status was ascertained.

The mean age of both cases and controls was 27.01 ± 3.06 years and 27.05 ± 3.43 years, respectively. Nearly similar proportions of cases (80.6%) and controls (82.3%) attended formal education (1–12 grade or above). Regarding religion, 93.5% of cases and 89.2% of controls were protestant. About 93.5% of cases and 90.6% of controls were Hadiya in ethnicity. About 60.2% of the cases and 59.9% of the controls had given birth two and more times (multiparous). Higher proportion of cases (71%) had a short inter-pregnancy interval than the controls (55.1%). Likewise, a higher proportion of cases (24.7%) had prolonged labor than the controls (8.6%) (Table 1).

**Table 1:** Socio-demographic, economic and reproductive characteristics of study participants with uterine atony in urban South Ethiopia, 2019–2020

Variables	Cases = 93 Number (%)	Controls = 372 Number (%)	Total (%) *N* = 465	X^2^ (*P*-value)
**Marital status**				
Married	92 (98.9)	368 (98.9)	460 (98.9)	1.00 (0.001)
Single/separated/divorced/widowed	1 (1.1)	4 (1.1)	5 (1.1)	
**Religion**				
Protestant	87 (93.5)	332 (89.2)	419 (90.1)	0.53 (2.22)
Orthodox	2 (2.2)	19 (5.1)	21 (4.5)	
Muslim	1 (1.1)	9 (2.4)	10 (2.2)	
Others^a^	3 (3.2)	12 (3.3)	15 (3.2)	
**Ethnicity**				
Hadiya	87 (93.5)	337 (90.6)	424 (91.2)	0.55 (1.18)
Kembata/Tembaro	4 (4.3)	18 (4.8)	22 (4.7)	
Others^b^	2 (2.2)	17 (4.6)	19 (4.1)	
**Education status**				
No formal education	18 (19.4)	66 (17.7)	84 (18.1)	0.13 (0.72)
Formal education	75 (80.6)	306 (82.3)	381 (81.9)	
**Occupation**				
Employed	15 (16.1)	71 (19.1)	86 (18.5)	0.43 (0.51)
Unemployed	78 (83.9)	301 (80.9)	379 (81.5)	
**Parity**				
1	37 (39.8)	149 (40.1)	186 (40)	0.002 (0.96)
≥ 2	56 (60.2)	223 (59.9)	279 (60)	
**Pregnancy intention**				
Intended	45 (48.4)	193 (51.9)	238 (51.2)	0.364 (0.55)
Unintended	48 (51.6)	179 (48.1)	227 (48.8)	
**Inter-pregnancy interval in months**				
< 24	66 (71)	205 (55.1)	271 (58.3)	7.69 (0.006)
24–60	27 (29)	167 (44.9)	194 (41.7)	
**Pre-eclampsia**				
Present	5 (5.4)	11 (3)	16 (3.4)	1.31 (0.25)
Absent	88 (94.6)	361 (97)	449 (96.6)	
**Premature rupture of membranes**				
Present	11 (11.8)	52 (14)	63 (13.5)	0.29 (0.59)
Absent	82 (88.2)	320 (86)	402 (86.5)	
**Progress of labor**				
Normal	79 (84.9)	316 (84.9)	395 (84.9)	0.001 (0.99)
Assisted	14 (15.1)	56 (15.1)	70 (15.1)	
**Duration of labor**				
Prolonged	23 (24.7)	32 (8.6)	55 (11.8)	18.56 (0.000)
Not prolonged	70 (75.3)	340 (91.4)	410 (88.2)	
**Mode of delivery**				
Spontaneous vaginal delivery	79 (84.9)	333 (89.5)	412 (88.6)	1.89 (0.39)
Cesarean section/Instrumental	14 (15.1)	39 (10.5)	53 (11.4)	
**Birth characteristics**				
Singleton	85 (91.4)	361 (97)	446 (95.9)	6.05 (0.01)
Multiple (twins)	8 (8.6)	11 (3)	19 (4.1)	
**Gestational age at birth in completed weeks**				
< 37 (pre-term)	15 (16.1)	39 (10.5)	54 (11.6)	7.86 (0.02)
37–41 (term)	65 (69.9)	307 (82.5)	372 (80)	
≥ 42 (post-term)	13 (14)	26 (7)	39 (8.4)	
**Birth weight in grams**				
< 4500	81 (87.1)	343 (92.2)	424 (91.2)	2.41 (0.12)
≥ 4500	12 (12.9)	29 (7.8)	41 (8.8)	
**Continuous variables**				
Age	27.01 (3.06)	27.05 (3.43)	27.04 (3.3)	–
Age at first childbirth	21.54 (2.76)	21.31 (2.59)	21.36 (2.6)	–

Key: Others^a^ = catholic, Apolostic, Joba witness; Others^b^ = Guragie, Siltie, Amhara, Tigray, Oromo, Wolayita; – not applicable

Data were missed for age (3) and age at first childbirth (1)

For continuous variables ‘mean (standard deviation)’ were estimated for each column

### Determinants of uterine atony

In the binary unconditional logistic regression model, five variables: the inter-pregnancy interval, duration of labor, birth characteristics, gestational age at birth, and birth weight, were associated with uterine atony at *P* < 0.20. When these variables were fitted in the multivariable unconditional logistic regression model, three variables: the inter-pregnancy interval, duration of labor, and birth characteristics, were found to be associated with uterine atony with a 95% CI at *P* < 0.05.

In this study, women with a short inter-pregnancy interval (< 24 months) were twice (AOR = 2.13, 95% CI: 1.26, 3.61) more likely to experience uterine atony than women with an inter-pregnancy interval of 24–60 months. This means that among women who had a short inter-pregnancy interval, about 53% of uterine atony was attributed to the short inter-pregnancy interval (AF = 53.05%, 95% CI: 20.63%, 72.29%). In the study population, about 38% of uterine atony was attributed to the short inter-pregnancy interval (PAF = 37.61%, 95% CI: 14.63%, 51.25%). Women who had a prolonged labor were twice (AOR = 2.35, 95% CI: 1.15, 4.83) more likely to experience uterine atony than those who had no prolonged labor. Among women who had prolonged labor, about 57% of uterine atony was attributed to the prolonged labor (AF = 57.44%, 95% CI: 13.04%, 79.29%). In the study population, about 14% of uterine atony was attributed to prolonged labor (PAF = 14.18%, 95% CI: 3.22%, 19.58%). Women who have given multiple birth (twins) were three times (AOR = 3.46, 95% CI: 1.25, 9.56) more likely to experience uterine atony than those with singleton birth. Among women who had multiple birth (twins), about 71% of uterine atony was attributed to multiple birth (AF = 71.09%, 95% CI: 20%, 89.53%). In the study population, about 6% of uterine atony was attributed to multiple birth (PAF = 6.11%, 95% CI: 1.72%, 7.69%) (Table 2).

**Table 2:** Multivariable unconditional logistic regression model for the determinants of uterine atony in urban south Ethiopia, 2019–2020

Variables	COR (95% CI)	*P*-value	AOR (95% CI)	*P*-value	AF (95% CI)	PAF (95% CI)
**Inter-pregnancy interval in months**						
< 24	1.99 (1.22, 3.26)	0.01	2.13 (1.26, 3.61)	0.004	53.05% (20.63, 72.29%)	37.61% (14.63, 51.25%)
24–60	1		1		1	1
**Duration of labor**						
Prolonged	2.49 (1.92, 6.33)	< 0.001	2.35 (1.15, 4.83)	0.018	57.44% (13.04, 79.29%)	14.18% (3.22, 19.58%)
Not prolonged	1		1		1	1
**Birth characteristics**						
Singleton	1		1		1	1
Multiple (twins)	3.09 (1.20, 7.93)	0.02	3.46 (1.25, 9.56)	0.016	71.09% (20, 89.53%)	6.11% (1.72, 7.69%)
**Gestational age at birth in weeks**						
< 37	1.82 (0.94, 3.50)	0.07	1.52 (0.75, 3.11)	0.25	–	–
37–41	1		1			
≥ 42	2.36 (1.15, 4.85)	0.02	1.88 (0.85, 4.15)	0.11	–	–
**Birth weight in grams**						
< 4500	1		1			
≥ 4500	1.75 (0.86, 3.59)	0.12	0.52 (0.15, 1.83)	0.31	–	–
Duration of labor*birth weight						
Prolonged Labor present * birth weight ≥ 4500 g	7.97 (1.29, 49.33)	0.03	10.38 (1.58, 68.13)	0.014	–	–

Keys: *AF* Attributable Fraction, *AOR *Adjusted Odds Ratio, *COR *Crude Odds Ratio, *PAF* Population Attributable Fraction, 1 = Reference category

## Discussion

This study aimed to identify determinants of uterine atony from cohorts of pregnant women who were followed-up until delivery. Accordingly, short inter-pregnancy interval, prolonged labor, and multiple (twin) birth were found to be determinants of uterine atony.

In this study, a short interval between pregnancies (< 24 months) was found to increase the risk of uterine atony by twofold as compared to 24–60 months of inter-pregnancy interval. The finding suggests that about 53% of uterine atony was attributed to short inter-pregnancy interval, which could be prevented if short inter-pregnancy interval was prevented among those women who had a short inter-pregnancy interval. Likewise, about 38% of uterine atony was attributed to short inter-pregnancy interval that could be avoided if the short inter-pregnancy interval was prevented in the study population. The impact of a short inter-pregnancy interval on the uterine atony could probably be due to the hypothesis that a short interval between pregnancies decreases the time to recover from previous abnormal uterine conditions such as incomplete healing of the uterine scars, abnormal process of remodeling of endometrial vessels, and nutritional depletion [32, 33]. A study conducted in Egypt [34] indicated that uterine atony-related postpartum hemorrhage was related to short inter-pregnancy intervals. The association between the inter-pregnancy interval and uterine atony has hardly been investigated, suggesting a need for further research. Inter-pregnancy interval is a modifiable condition that can be improved by increasing the use of modern contraceptive methods in the community, mainly by increasing the utilization of long-acting modern contraceptive methods since they can give longer protection [35].

Women who had prolonged labor (labor that exceeds 12 h) were twice more likely to experience uterine atony than those who had no prolonged labor. The result indicates that about 57% of uterine atony was attributed to prolonged labor, which could be prevented if prolonged labor did not happen. Similarly, about 14% of uterine atony was attributed to the prolonged labor that could be avoided if prolonged labor did not happen in the study population. This might be due to the fact that when women stay in labor for longer durations (often due to obstructed labor), the uterine muscle (myometrium) contracts several times to deliver the baby, and the blood vessels are also compressed together with the uterus. Following the delivery of the baby, the uterine muscle and the blood vessels delay to return back to their normal state through contraction, instead remain relaxed and soften resulting in uterine atony [5, 10]. The evidence is supported by the studies conducted in Pakistan and Madagascar, where prolonged second stage of labor increased the odds of uterine atony [11, 36].

The risk of uterine atony was higher for women who had multiple (twin) birth as compared to singleton birth. Although multiple pregnancy cannot be modified or prevented, the result of this study suggests that women with multiple pregnancies should be given due attention to reduce the risk of uterine atony. The association seems physiologically plausible; when multiple pregnancies occur, the uterine wall becomes overly distended during pregnancy and the uterine muscles relax for a longer duration. This condition is further aggravated during labor when the uterine muscles contract and relax to deliver the baby [5]. However, after delivery, the overly distended and stretched uterus fails to contract adequately, which leads to uterine atony. The finding was consistent with other studies [3, 10],

This study further revealed that there was interaction (possible effect modification) between prolonged labor and birth weight ≥ 4500 g (macrosomia). According to the result, prolonged labor and birth weight ≥ 4500 g jointly increased the risk of uterine atony by tenfold, which suggests the need for due attention for those women with prolonged labor and babies with macrosomia.

Despite the attempts made to reduce it, this study might have the following limitations: First, in a nested case–control study, cases and controls originate from cohort studies. Thus, it provides useful information about temporal relationships that conventional case–control studies cannot. However, selection bias and loss of power are common concerns [37–39]. Selection bias might have occurred as this study was a subset of the cohort study and based on the available number of cases and corresponding controls. Selection bias might have also occurred, as some women might not have been included during recruitment due to the fact that they are unable to remember the date of the last childbirth, even though we have involved family members such as the husband, older children, and mother-in-law, birth date ceremonies and tried to see immunization cards. To some extent, selection bias can be minimized by taking controls from cohorts’ risk sets using random sampling techniques, especially when the cohort study is from a defined population. Likewise, increasing the number of controls per case may help increase the power somehow. Thus, we attempted to increase the sample size by increasing the number of controls per case to four. Second, recall bias might have occurred related to recalling the date of the last menstrual period, although we tried to minimize it by considering the ultrasound scan. Third, intraobserver bias might have happened while performing an ultrasound scan by the person who estimated the gestational age for those women who had difficulty recalling the LMP. Regardless of the limitations, the findings of this study will provide useful information for decision-making in clinical practice and can be generalized to similar populations in similar contexts.

## Conclusions

Uterine atony was related to mostly modifiable conditions that could be improved by increasing the utilization of maternal health services such as modern contraception, antenatal care, and skilled birth attendance in the community. Creating awareness among couples and the community at large about the importance of adequately spacing pregnancies using modern contraceptive methods, timely referral of women when labor starts, and giving due emphasis to women with multiple pregnancies need to be underlined to prevent uterine atony and related maternal morbidity and mortality. Further research is recommended to assess the untoward outcomes of uterine atony.

## Supplementary Information

**Additional file 1: Figure 1.** Flow-diagram of the overall study process in urban South Ethiopia, July 2019–September 2020.

## Data Availability

The raw materials that support the conclusions of this research will be available to researchers, who need the data to use for non-commercial purposes through requesting the corresponding author.

## Abbreviations

AF: Attributable Fraction
AOR: Adjusted Odds Ratio
COR: Crude Odds Ratio
LMP: Last Menstrual Period
PAF: Population Attributable Fraction
WHO: World Health Organization
