# Supplementary material for: Determinants of postpartum uterine atony in urban South Ethiopia: a community-based unmatched nested case–control study
Source: BMC Pregnancy Childbirth. 2023 Jul 6;23:499. doi: 10.1186/s12884-023-05820-1 (PMC10326949; doi:10.1186/s12884-023-05820-1)
Supplement: Supplementary file 1 — Additional file 1: Figure 1. Flow-diagram of the overall study process in urban South Ethiopia, July 2019–September 2020. [file 12884_2023_5820_MOESM1_ESM.doc]

Additional figure 1.

| South Region, Ethiopia  Hadiya Zone town administrations **(5)**  Pregnant women eligible for the study  **(2629)**  Pregnant women eligible for baseline interview and followed up **(2578)**  Refused to participate **(51)**  Not wanted to be followed =10  Not wanted to mention reason=41  Loss of follow up **(29)**  Study period ended= 21  No information at all =8  Women whose pregnancy outcomes were ascertained at the end-line and included in the analysis **(2548)**  Spontaneous abortion (<28 weeks) **(1)**  Pregnancy end without uterine atony  **(2455)**  Women whose pregnancy outcomes were ascertained **(2549)**  Uterine atony  **(93)**  Additional figure 1. Flow-diagram of the overall study process in urban South Ethiopia, July 2019–September 2020. |
| --- |
